# Addressing Teamwork Delays during Life-Saving Interventions through an Activity Theory-Informed Analysis

**DOI:** 10.1145/3701195

**Published:** 2025-01-10

**Authors:** KATHERINE ANN ZELLNER, ALEKSANDRA SARCEVIC, MAJA BARNOUW, MEGAN A. KRENTSA, TRAVIS M. SULLIVAN, MARY SUHYUN KIM, RANDALL BURD

**Affiliations:** Drexel University, USA; Drexel University, USA; Drexel University, USA; Drexel University, USA; Children’s National Hospital, USA; Children’s National Hospital, USA; Children’s National Hospital, USA

**Keywords:** Activity theory, complex teamwork, video review, computerized support, delay mitigation, hemorrhage control, trauma resuscitation

## Abstract

Hemorrhage, or severe blood loss due to injury, is a leading cause of preventable deaths after injury. This study uses and extends activity theory to understand the dynamics of team-based hemorrhage control during trauma resuscitation and to explore potential computerized mechanisms to support this time- and safety-critical process. We reviewed videos of 25 resuscitation cases and analyzed hemorrhage control activities using nine activity theory prompts, including a new prompt—speech intention—a critical but underexplored dimension of teamwork in prior activity theory analyses. Through this process, we identified the most common delay-causing activities and developed routine and non-routine activity models for each. A comparison of these models showed that variations from the routine models emerged due to changes in the division of labor, instruments, community, and speech intentions. We contribute to research on designing socio-technical systems by (1) identifying needs and opportunities for computerized support that address delays in complex medical teamwork and (2) examining how an intervention changes an activity model. We also show how adding detailed speech data aids in identifying contradictions between elements in an activity model.

## 1 Introduction

In high-stakes environments, effective teamwork is critical in mitigating adverse outcomes [4]. The field of computer-supported cooperative work (CSCW) has long studied these dynamic scenarios, seeking to understand and enhance collaborative efforts through computerized interventions in a range of domains [33][34][48][54]. Managing hemorrhage is an example of a critical, high-stakes task that embodies the challenges and requirements of a rapid and coordinated team response. Hemorrhagic shock, or the physiologic instability associated with severe blood loss, is the leading cause of preventable deaths after injury [1]. Early recognition and intervention of hemorrhagic shock are associated with decreased mortality and morbidity, including fewer hospital-acquired infections and shorter hospital stays [19][44][59]. Reducing the time to blood transfusion is critical to improving patient survival because every minute delay increases the mortality rate by 5% in patients who require massive blood transfusions [44]. To minimize transfusion delays, recent interventions have focused on enhancing the accessibility of blood products [50][51]. These efforts, however, target instrument- and resource-based challenges rather than skill-based or decision-making errors associated with major disruptions in hemorrhage control and surgical workflows [20][36]. Addressing delays due to skill-based errors requires an in-depth study of hemorrhage control activities from a sociotechnical perspective.

Using conceptual frameworks to understand medical teamwork can help improve clinical outcomes [56]. In HCI and CSCW research, activity theory-informed analyses are often used to understand complex processes and inform technology design [22][38]. This descriptive theory explains how people achieve goals through the mediation of artifacts, social contexts and rules, and division of labor [45]. In healthcare, activity theory can be used as an analytic lens to understand the complex relationships between patients and clinicians and propose guidelines for developing future interventions [8][13][27][39][40].

This paper uses an activity theory-informed analysis to examine hemorrhage control during trauma resuscitation not only as a medical emergency but also as a team-based process where socio-technical approaches and interventions can influence outcomes. Hemorrhage control consists of several activities, including the identification of hemorrhage, the decision to transfuse blood, and the initiation of blood transfusion [15]. Because this process involves multiple steps and many participants, it is well-suited for an activity theory-informed analysis. We pursued three research goals: (1) identify activities in the hemorrhage control process that can most benefit from computerized support, (2) determine potential mechanisms for computerized decision support, and (3) examine the effects of an intervention on an activity theory-informed model of a hemorrhage control activity. To achieve these goals, we analyzed videos of 25 trauma resuscitation cases that included hemorrhage control. For the first two goals, we used 17 cases to identify delays in care and then responded to eight activity theory prompts for each activity [45]. We also explored the impact of speech on activity models by associating transcribed speech lines from resuscitation videos with each activity and determining speech intentions. We standardized all prompt responses and developed generalized activity models for four activities that emerged as the most frequent causes of delays: (1) establishment of vascular access, (2) decision to transfuse blood, (3) acquisition of blood products, and (4) setup of the rapid infuser. For the first three activities, we found two contrasting activity theory models: a *routine model* with less frequent delays, and a *non-routine model* associated with increased delays. By focusing on the “contradictions” [38] that emerged from comparing routine and non-routine models, we identified several design opportunities. The absence of contradicting models for the rapid infuser setup activity highlighted issues in the physical aspects of the activity, suggesting non-computerized solutions. The introduction of a blood refrigerator to address delays in obtaining blood products during the study period allowed us to achieve our third goal and examine the extent to which an intervention changes an activity model. We reviewed eight additional resuscitation cases that occurred after the refrigerator was introduced. We developed new, post-intervention activity models for the “acquisition of blood products” activity and compared them with the pre-intervention models.

We make two contributions to research on designing socio-technical systems: (1) identifying the needs and opportunities for computerized support that addresses delays in complex medical teamwork and (2) demonstrating how a low-tech intervention affects an activity model to lay the foundation for future use of activity theory as a tool in assessing the impacts of clinical interventions. We also propose including speech data and speech intentions in activity theory analyses for a more comprehensive understanding of complex teamwork in different contexts.

## 2 Related Work & Research Context

Below, we review two related areas of research to highlight our contributions. First, we discuss prior studies on delays and process deviations in medical settings. We then review prior research that used activity theory to inform technology design. We conclude with a brief overview of the hemorrhage control process.

### 2.1 Delays and Process Deviations in Medical Processes

Prior medical studies have investigated process deviations and their effects on patient care during surgery [29][37][51], postoperative care [62], and trauma resuscitation [36][61][63]. Process delays are usually described as non-routine events leading to workflow disruptions [15]. Several studies have identified the causes of delays through in-situ observation [36][52] and video review [29][61]. For example, Parker et al. [52] attributed delays during surgical procedures to technical factors, environment, training and procedures, and teamwork. Another study of surgical workflow disruptions found that communication and coordination problems were the most frequent contributors to provider and organizational delays [29]. Communication issues were often due to failures to understand requests for information. Coordination problems arose from absent personnel, causing delays and incorrect decisions made in their absence. To address delays, team members made compensatory actions, such as increased efforts in improving team communication and task coordination. For example, when a team member communicated a patient’s critical status to the surgeon, they promptly engaged in problem solving [29].

The most common factors associated with process deviations in trauma resuscitation include equipment malfunction, aberrant activities by personnel, patient factors, interruption, and external and family action [63]. Trauma teams acknowledged the deviations 78% of the time and performed compensatory actions for 69% of deviations. The teams’ response to errors also varied based on error type, risk, resuscitation phase, and mechanism of injury [63]. For example, to compensate for errors of omission, like failing to stabilize the cervical spine, the teams repositioned the cervical collar, mitigating potential harm. Similarly, errors of commission, like performing an incorrect procedure, were frequently corrected by returning to the protocol, while high-risk errors, such as undetected irregular heart rate, prompted immediate interventions upon acknowledging the error. A more recent analysis of 12 trauma resuscitation cases identified several factors associated with delays specific to hemorrhage control, including the need to deliver blood to the emergency department, provider inexperience in using the rapid fluid infuser, difficulty in establishing vascular access, and provider indecisiveness [60].

This prior work identified the types of delays, process deviations, and most common contributors to delays. However, our understanding of how teams manage delays and how non-routine events affect teamwork remains limited. In this paper, we focus on understanding the performance of medical teams during hemorrhage control and examining how delays impacted their collaboration.

### 2.2 Activity Theory and its Applications in CSCW and Medical Research

Complex teamwork within different contexts has been studied in CSCW using several theoretical frameworks, including activity theory, distributed cognition, and situated action models [8][22][28][46]. Activity theory emerged from the Russian school of psychology during the 1920s and 1940s. Engeström expanded the initial framework by including elements that reflect collective and collaborative nature of human activity [16][17]. This framework emphasizes the integration of individual roles and motivations, artifacts, and contextual elements, allowing for a comprehensive understanding of how individual roles and tools contribute to the overall activity [16][17]. Distributed cognition is a cognitive science theory focused on the interaction between various components within a cognitive system composed of individuals and artifacts [28]. While useful for understanding the collective aspects of an activity and its completion, it may not fully capture the individual roles and motivations that drive specific actions within the system. Situated action models prioritize the real-time, responsive nature of actions, highlighting how goals often emerge retrospectively [46]. Although situated action models account for the dynamic nature of a complex medical process, they may not adequately address the predefined protocols and intentions inherent in these scenarios.

We selected activity theory as our analytic approach because it provided a more holistic and contextually grounded framework for understanding the hemorrhage control process from a socio-technical perspective. Based on Engeström’s triangle, each model has six interactive components: subjects, objects, tools/instruments, rules, community, and division of labor [17]. The subject can be an individual or a group with a shared objective. Subjects engage in multiple activities with distinct objectives. Tools include the artifacts that subjects use to carry out activities. Rules include explicit or implicit routines, policies, guidelines, and rituals that govern and regulate activity within the community. Division of labor describes how tasks and responsibilities are distributed among community members. The community involves all subjects within an activity and their environment. Rules mediate between subjects and the community, influencing the overall functioning of the community, while the division of labor determines how the community collectively works toward the shared objective [17]. Mwanza built on Engeström’s triangle by creating the AODM activity theory framework, an eight-step approach for modeling how an activity is achieved through answering eight prompts [45]: (1) the activity of interest (activity), (2) the reason for the activity (object-ive), (3) the roles involved in the activity (subjects), (4) the tools (instruments) used to perform the activity, (5) the routines, policies, and other formal or informal rules that influence the activity (rules), (6) the roles and responsibilities of each subject (division of labor), (7) the nature of the community and environment (community), and (8) the desired outcome from carrying out the activity (outcome). In this study, we used this AODM activity theory framework to analyze the effects of delays on trauma teamwork during hemorrhage control.

Activity theory has been used for studying different practices in healthcare settings, including planning and collaboration [3][5][22], diagnostic processes [17], surgical coordination [6], and chronic illness management [27]. For example, activity theory was used to investigate the coordination and planning of patient care within hospitals, informing the design of a computer-based patient scheduler [3]. Cornet et al. [13] also applied Mwanza’s activity theory framework [45] to understand how patients with heart failure manage their condition and communicate with community members. More recently, activity theory has also been used to determine the impact of interventions within the classroom setting [9][23][30]. To identify areas for improvement, these studies focused on identifying “contradictions” (the force behind the development of an activity) between the elements in the activity system because contradictions tend to improve the activity after being resolved [38]. For instance, integrating activity theory into analyses of interviews with students and instructors helped highlight the contradictions within a graphic design portfolio activity [42]. We contribute to this body of work by using activity theory to identify design opportunities for improving a complex medical process. In addition, our activity theory analysis is based on video review. Unlike interviews and participant recollections obtained through participatory design approaches (e.g., [5][26][41]), videos capturing real-time activity from multiple angles offer a more nuanced and detailed view of actions, interactions, and processes.

#### 2.2.1 Speech as an Additional Activity Theory Element:

Although speech plays an important role in achieving a team-based activity, it has not been formally represented in activity theory as a prompt in the analysis. Rather, it is implicitly implied through other activity theory prompts, such as environment, division of labor, and community. Studying team-based speech during an activity can help us understand how a team achieves the activity goals. During trauma resuscitation, speech is used to coordinate and communicate information through information requests, progress reports, task assignments, and confirmations. Speech in this domain is also an example of articulation work, the process of organizing tasks within teams and facilitating the flow of work [57][58]. Prior work on using speech in resuscitation settings classified speech to represent the sequence of team activities [55]. A subsequent study expanded this classification into speech intentions to determine the role of speech in detecting delays during life-saving interventions [67] (Table 1). In this paper, we used videos and transcripts of resuscitation cases, which allowed us to associate speech lines with different activities in hemorrhage control during the activity theory analysis and identify speech intentions and patterns across activities and models. By considering detailed speech data in our analysis, we showed how speech intention could be used to distinguish between routine and non-routine activity models. Our results suggest that adding speech intention as a prompt can affect activity theory-informed analysis and its findings.

### 2.3 Research Context: Hemorrhage Control

Hemorrhage control provides an ideal context for studying teamwork delays because it involves multiple steps and many participants. To rapidly evaluate and manage severely injured patients, trauma resuscitation teams follow a two-part protocol [1]. The first part (primary survey) is designed to identify and address immediate life-threatening injuries, including injuries to the airway, respiratory, circulatory, and neurological systems. The second part (secondary survey) is designed to evaluate the patient from head to toe for all other injuries. Hemorrhage control falls within the circulation step of the primary survey and involves identifying and stopping uncontrolled bleeding, diagnosing hemorrhagic shock, and transfusing blood. The process can be summarized into seven activities [15] (Table 2). These activities typically occur in the listed order, but some may be simultaneous or repeated multiple times.

## 3 METHODS

We conducted our study at a Level 1 pediatric trauma center of an urban teaching hospital in the mid-Atlantic region of the United States. On average, the center treats about 600 trauma patients each year. The emergency department has two trauma bays, each equipped with necessary instruments, supplies, and equipment (e.g., vital sign monitors and surgical supplies). Each trauma bay also has an always-on video and audio recording system that activates when motion is detected in the room. The cameras are positioned at different angles to capture the views from above the patient bed and at the foot of the bed, where the leadership team assembles to guide the resuscitation. The audio recording system includes directional microphones above the bed and the leadership team. At our research site, all trauma resuscitations are co-led by a surgical team leader (senior surgical resident or surgical fellow) and an emergency department (ED) physician. Their role is to obtain and interpret information received from the bedside physicians and nurses who provide hands-on patient care and to make decisions. The hospital’s Institutional Review Board (IRB) approved this study. All data used in the study were protected and secured according to the IRB policies and the Health Insurance Portability and Accountability Act (HIPAA).

### 3.1 Data Selection

To select cases for our analysis, we drew from 1,704 trauma resuscitations that occurred at our research site between March 2018 and June 2021. Our inclusion criteria were: (1) the patient received blood in the trauma bay, (2) video and audio recordings were available for analysis, and (3) research participation consent was obtained from a parent or caregiver. Seventeen cases matched these criteria. To examine the effects of an intervention on an activity model (our third research goal), we also included cases occurring after a blood refrigerator was installed in the trauma bay in June 2021 to reduce delays in obtaining blood products. The trauma team had access to the blood refrigerator in eight cases. The 25 total cases included 163 instances of hemorrhage control activities, providing a sample size appropriate for generating meaningful insights in a qualitative study [9].

Using the video and audio recordings of the selected cases, we manually generated transcripts based on a pre-determined protocol to ensure consistency. First, the transcribers censored any identifiable data from the recordings to preserve patient and provider confidentiality. They then listened to the audio recordings to transcribe speech utterances and speaker roles and add timestamps for each utterance. Speaker roles were determined based on the speech content (e.g., bedside physicians report the examination results, leaders make decisions), differences in speaker tones, and transcriber’s domain knowledge. The transcribers repeatedly listened to the audio file to correctly transcribe overlapping speech lines from several speakers, leading to about 30 minutes of transcription time per 10-minute segment. The average case duration was 32.6 minutes (M = 35.29 minutes, SD = 17.7 minutes).

### 3.2 Data Analysis

We prepared and analyzed our data in six steps. First, a trauma surgeon identified process delays in all cases. Second, we determined the hemorrhage control activities in each case, responded to the eight prompts from the AODM activity theory framework, and determined the speech intention for each activity [45]. Third, we reviewed all activity theory prompt responses and performed the first language standardization round before building models for each activity. Fourth, we conducted a second round of standardization and further model generalization. In the fifth step, we established generalized activity theory models for four hemorrhage control activities that emerged as the most frequent causes of delays. Finally, we analyzed eight additional cases after the blood refrigerator was introduced to improve the “acquisition of blood products” activity.

#### 3.2.1 Step 1: Identification of Process Delays:

A trauma surgeon on our research team reviewed video recordings of all cases, identifying process delays and their potential causes using a previously established protocol. The surgeon also recorded timestamps to mark the start and end times of each delay and its resolution. A delay was recorded if: (1) team members had moved on to the next phase of the protocol without completing the activities in the earlier phase, (2) a team member needed a prompt to start an activity, (3) a team member waited for the completion of the preceding activity before starting their activity, and (4) a team member executed an activity with pauses, and needed a prompt to continue or speed up the performance. Through this analysis, we identified 43 separate delays and their causes across the 17 cases. These data were recorded separately from the activity theory coding to avoid influencing the process. Because our long-term research goal is to address delays in blood transfusion by reducing communication lapses and the time to intervention, we focused on identifying delays in processes and procedures rather than in decision making.

#### 3.2.2 Step 2: Activity Theory Coding:

Two medical students with knowledge of trauma resuscitation reviewed the video of each case, marking hemorrhage control activities and their start and end times in a worksheet. Through this process, we identified 148 instances of hemorrhage control activities in the 17 cases. For all 148 activity instances, we then entered responses to the activity theory prompts, identifying the subjects, object(ive), instruments, division of labor, outcome, community, and rules. For subjects, we determined the trauma team roles involved in the specific activity. To answer the object(ive) prompt, we identified the goal of the activity. For instruments, we identified any artifacts used to complete the activity. In response to the division of labor prompt, we described the responsibilities of each team role involved in the activity. For outcome, we provided the expected result for each activity. For community, we described the conditions in the room (e.g., noisy, quiet, chaotic). For rules, we identified the protocols followed during the activity. In addition to these activity prompts, we identified speech utterances associated with each activity from the transcripts and labeled them with the speech intention. At the end of this step, each of the 148 activity instances had a unique set of activity theory analysis responses.

#### 3.2.3 Step 3: Activity Theory Response Review and First Round of Language Standardization:

We next reviewed all 148 activity theory responses and performed the first round of language standardization to facilitate model development and generalization for each activity. This process focused on standardizing the terms used in each response while maintaining significant differences between the prompts and ensuring that descriptions were functionally similar within each activity. For example, the goal for “blood transfusion” activity in one case was described as “the patient’s hemodynamic status improves,” while for another was “patient receives fluid to improve unstable volume status.” Because these two responses were clinically similar, we standardized the language for this prompt response to “improve the patient’s hemodynamic status.” This standardization step initially resulted in 57 different activity theory model variations across all activities (Table 3: Initial AT Models).

#### 3.2.4 Step 4: Second Round of Standardization and Activity Theory Model Building:

We performed the second round of standardization to further generalize the activity models, consulting a surgeon to ensure that we preserved distinctions in clinical processes, descriptions, and outcomes. We standardized the language within each activity and across all activities to facilitate model comparisons. For example, “calm but busy trauma bay” and “busy trauma bay” were synthesized into “busy trauma bay.” We also combined attending physician and fellow roles into “team lead (attending/fellow),” and different nurse roles into “nurses.” Through this second round of standardization, we reduced the number of activity models from 57 to 36 across all activities (Table 3: Standardized AT Models).

#### 3.2.5 Step 5: Activity Theory Analysis:

Our objective in this step was to establish a generalized activity theory model for the activities that most frequently caused delays in hemorrhage control. We started by reviewing 36 model variants that emerged from the previous step. We excluded from further analysis three activities that did not cause delays in hemorrhage control: patient arrival, hemorrhage identification, and blood transfusion (Table 4: Delay Causing). The remaining four activities—the decision to transfuse, the establishment of vascular access, the acquisition of blood products, and the setup of the rapid infuser—were frequently delayed and caused most delays in subsequent activities. We reviewed and discussed the 25 standardized models from the four activities, identifying the commonalities and differences between the elements within each activity. We observed that the models could be categorized into “routine” and “non-routine” scenarios based on the elements and variations within each category. As a result, we synthesized routine and non-routine models and created generalized representations for each delay-causing hemorrhage control activity.

#### 3.2.6 Step 6: Pre- and Post-Intervention Activity Theory Model Comparison:

Using the eight post-intervention cases, we developed the generalized activity model representing the eight “acquisition of blood product” activity instances in which the blood refrigerator was available in the trauma bay. We compared this model to both routine and non-routine pre-intervention models of the same activity. Our comparison focused on the contradictions between the elements across the three activity models.

## 4 FINDINGS

We report our findings in three parts. First, we present the findings from the delay analysis of each hemorrhage control activity and the factors associated with delays. Second, we describe the activity models from four delay-causing hemorrhage control activities. After describing each model, we highlight the effectiveness of adding speech intention and how this prompt contributed to the descriptive power of the model. Finally, we present the findings from the comparison of pre-and post-intervention activity models for the “acquisition of blood products” activity.

### 4.1 Delays in Hemorrhage Control and Associated Factors

Across the 17 cases, we identified 43 unique delays during hemorrhage control. The most common activity leading to delays was the rapid infuser setup. Clinicians had difficulty setting up the infuser and preparing blood for transfusion in 14 instances (Table 4: Delay Causing). The next most common delay-causing activity was the acquisition of blood products. Delays in accessing blood were observed in 12 instances and often led to further delays in subsequent hemorrhage control activities. The team was delayed in establishing vascular access and deciding to transfuse blood in nine instances. These delays may be linked to the longer durations of non-routine activities when compared to routine activities (Table 4).

### 4.2 Activity Theory Models for Four Delay-Causing Hemorrhage Control Activities

We developed seven generalized models across the four delay-causing hemorrhage control activities (Table 3: Generalized AT Model Variations). For transfusion decision, establishing vascular access, and obtaining blood products, we found two contrasting activity theory models: (1) the *routine* model that represented the most common activity performance and had fewer delays, and (2) the *non-routine* model that diverged from the routine performance and was associated with increased delays. For the rapid infuser setup, we found one activity model. Below, we describe each activity, highlighting the differences and commonalities between the routine and non-routine models.

#### 4.2.1 Establishment of Vascular Access:

**Figure 1:**
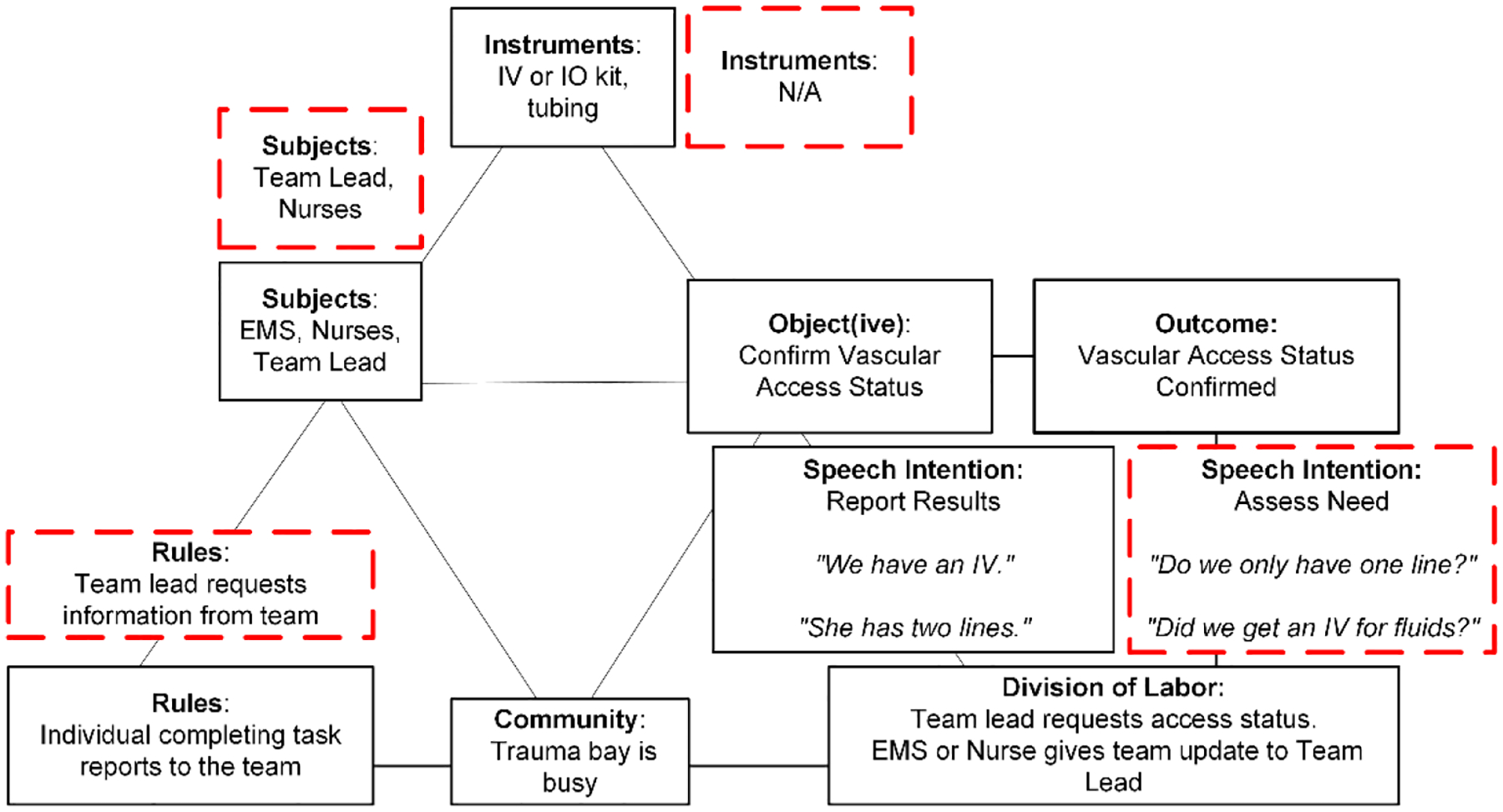
A graphical representation of the activity model for the “establishment of vascular access” activity. The routine model is shown in the solid black boxes while deviations seen in the non-routine model are shown in the red-dashed boxes.

In the routine model, the primary subjects are emergency medical services (EMS) personnel transporting the patient to the hospital, nurses, and the team lead (Figure 1). The object(ive) is to either establish vascular access or confirm the status of previously established access. The team achieves this goal by using instruments such as an intravenous (IV) or intraosseous (IO) kit and tubing, or by visually assessing the patient. When a patient arrives with established vascular access, the EMS team informs the trauma team about the IV status. The team lead and nurses listen to the report and then confirm the status. When a patient does not have established vascular access upon arrival, a nurse works to establish access, sometimes with the assistance of another nurse. Regarding the rules prompt, the routine model follows a report-based communication protocol, where each team member reports out loud the status and results of their tasks. The community is typically described as busy—everyone appears to be going about their tasks, rapidly moving around the patient, and completing their work. Speech intentions are most often “report results” (Table 1), exemplified by phrases like “We have an IV,” or “She has two lines.” The desired outcome is the confirmation that vascular access is established.

The non-routine model for establishing vascular access departs from the routine model in four elements: the subjects include only the team lead and nurses, no instruments are used, the rules shift to an assessment-focused interaction, and speech intentions shift to “assess need for an activity.” These shifts reflect a transition from certainty in the routine model to uncertainty in the non-routine model. The team lead in the non-routine model must actively assess the need for vascular access and engage in discussions. Example utterances include questions such as “Do we only have one line?” or “Did we get an IV for fluids?”

One delayed case that aligned with the non-routine model had issues in communicating and establishing access. The patient arrived with functioning IO access, following the routine model as the EMS team reported the status of vascular access to the team lead. Later in the case, we observed a long discussion between team members assessing the need for secondary access to administer medication. After deciding to proceed with another access point, the team spent nearly 30 minutes attempting to establish this second access. The delay in deciding about the second access point and the unsuccessful attempts to establish access caused further delays in transfusing blood.

For this activity, speech—marked by an increase in team discussions—helped distinguish between the routine and non-routine cases. The shift in speech intentions highlighted the team’s shift from certainty to uncertainty around IV access. The other elements in the model, such as instruments or subjects, showed limited signs of this transition. A model of this activity without the speech element would have limited our insight into how teams navigated uncertain situations, with the changes in rules serving as the central indicator.

#### 4.2.2 Decision to Transfuse Blood:

**Figure 2:**
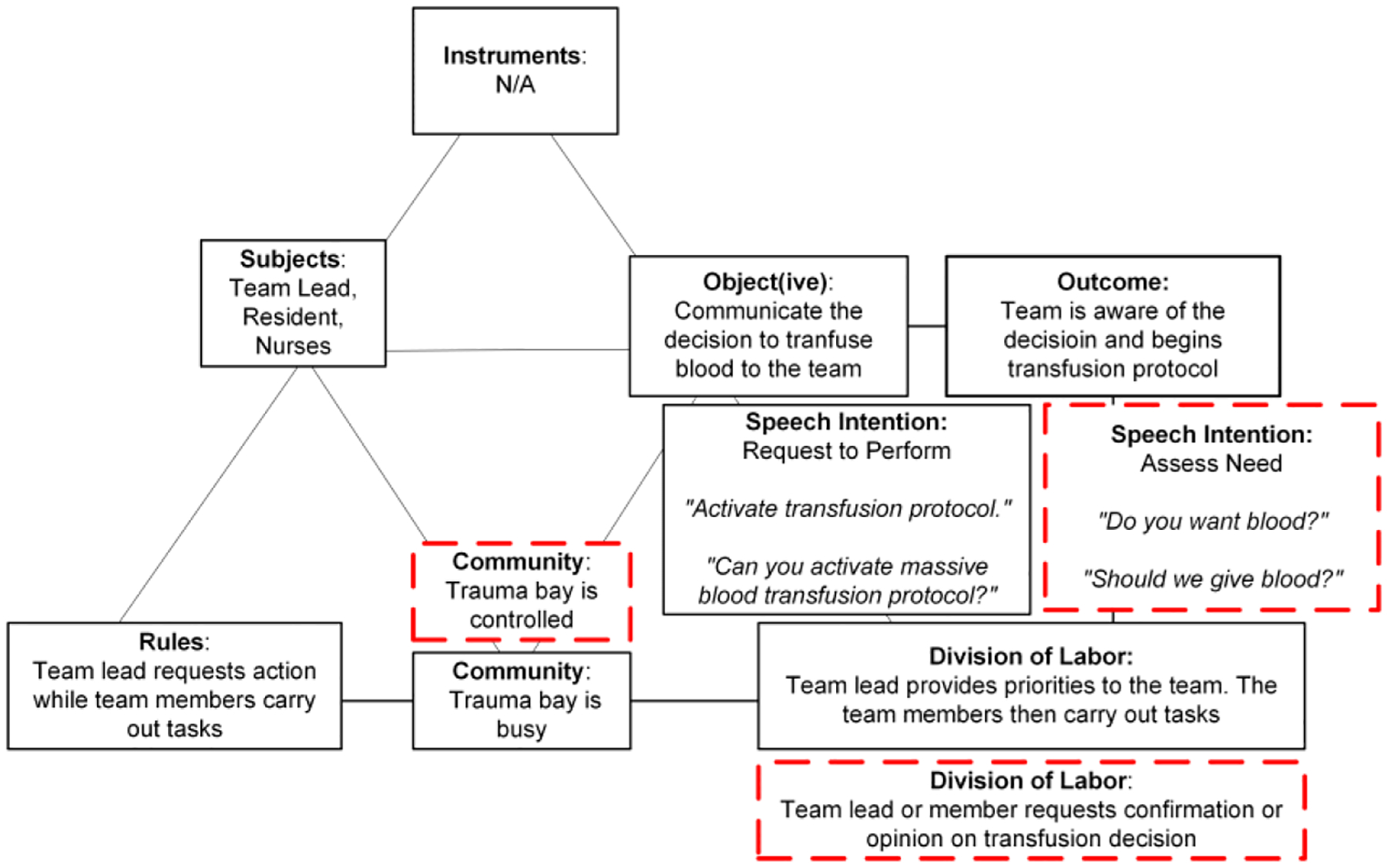
A graphical representation of the activity model for the “decision to transfuse blood” activity. The routine model is shown in the solid black boxes while deviations seen in the non-routine model are shown in the red-dashed boxes.

In the routine model, the subjects making decisions are the team lead and nurses (Figure 2). The object(ive) is to effectively communicate this decision and ensure the awareness of the entire team. No specific tools or instruments are used in this activity. The team lead provides priorities and communicates the decision (e.g., “I think that we will need to give blood here.”). In contrast, the individual team members are responsible for executing the associated tasks (e.g., prepare equipment). The rules governing this activity dictate that the team lead initiates the decision-making process and team members execute the tasks. The community is busy but orderly. Speech intention is mostly “request to perform,” with the team lead giving directives like “Activate transfusion protocol,” “Can you activate massive blood transfusion protocol?” or “Is someone available to begin infusion protocol? We’ll start with O negative blood.” The desired outcome in this model is the team’s awareness and understanding of the transfusion decision.

The non-routine model for transfusion decision departs from the routine model in three elements: speech intentions shift towards “assessing the need” for blood transfusion, the room (community) is calmer as team members engage in discussion, and the division of labor changes as the team lead and other members actively consider blood transfusion and seek each other’s opinions. These shifts reflect uncertainty expressed through inquiries directed to the team lead: “We don’t need blood… Do we need it?” and “Do you [team lead] want blood?” or “Should we give blood?” The questions indicate that the decision to transfuse has not been made or it has not been effectively communicated to the team.

A delayed case that followed the non-routine model had patient severely injured by a gunshot wound. The team lead announced an anticipated poor outcome. Before the patient arrived, a nurse assessed the need for blood, asking the team lead: “We don’t need blood? Do we need it? Do we?” After the patient arrived and the team began stabilizing the patient, team members were still uncertain of the priorities. A team member asked, “would you prefer blood to saline?” twice before the team lead finally made the decision and responded, “let’s set up blood.” In contrast, in another case with a severely injured patient by a gunshot wound, the team lead began the case by clearly communicating the decision and delegating tasks to team members: “We’re gonna do a rapid infusion of blood. So, whoever is in charge of getting all that, please go ahead and set up the infuser.” The team in this case did not experience any delays, suggesting that clearly communicated decision allowed team members to start their tasks and prepare for blood transfusion in a timely manner.

Speech again played a critical role in identifying the differences between routine and non-routine models. In the routine model, communication is directive and task-focused, supporting straightforward activity execution. However, in the non-routine model, speech becomes a tool for consensus-building, reflecting a shift in how decisions are approached and finalized. Without seeing this change in verbal communication patterns, we would have missed the main difference between the routine and non-routine models.

#### 4.2.3 Acquisition of Blood Products:

**Figure 3:**
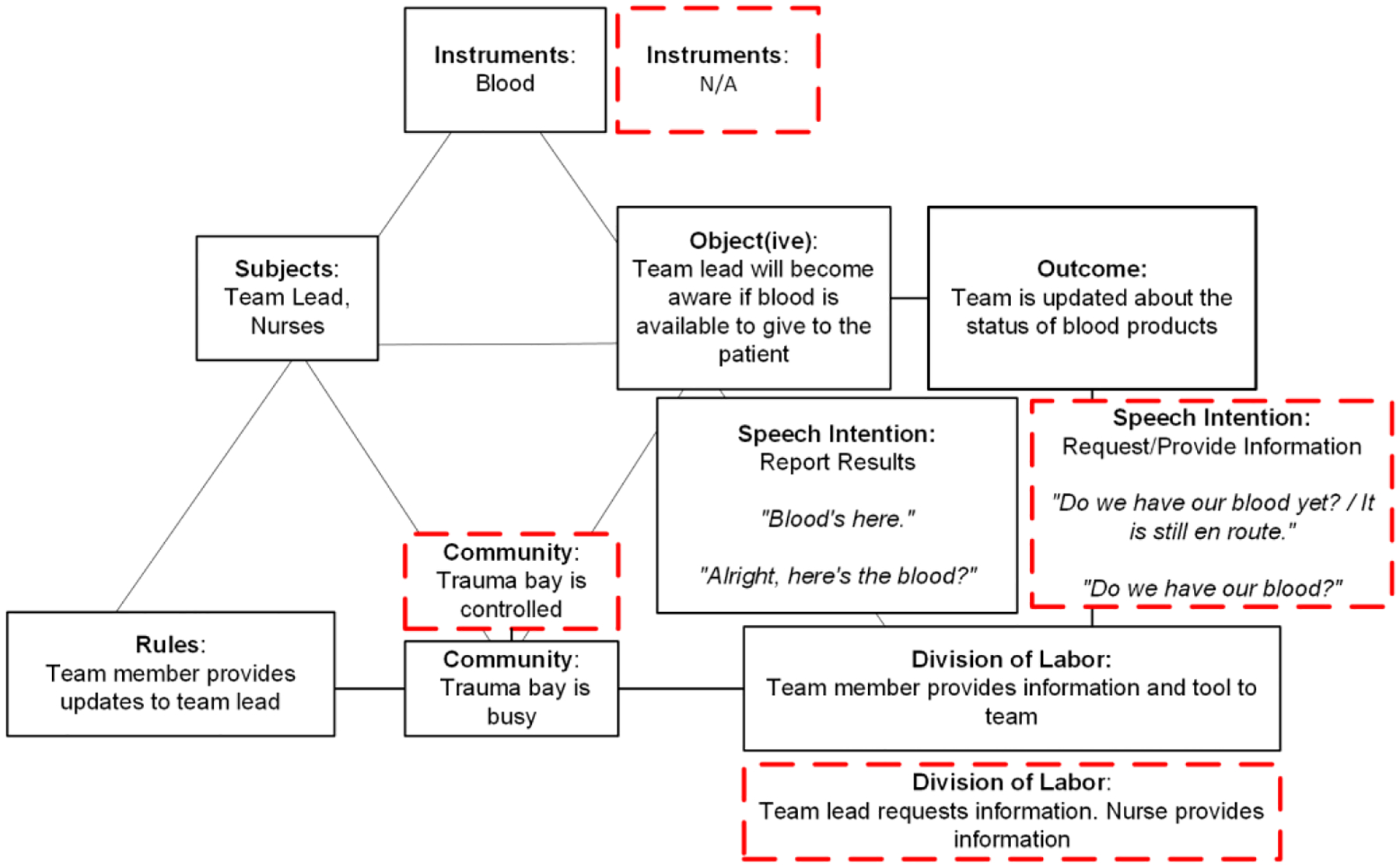
A graphical representation of the activity model for the “acquisition of blood products” activity. The routine model is seen in the solid black boxes whereas deviations seen in the non-routine model are in the red dashed boxes.

In the routine model, the subjects are the team lead and nurses (Figure 3). The object(ive) is to inform the team lead when the blood products are obtained and ready for administration. The instrument is blood products. The division of labor is straightforward: a staff member delivers the cooler with blood products to the team, followed by a team member informing the team lead and the rest of team that blood has arrived. The rules for this activity dictate that team members inform the team lead when the blood products arrive. The community is busy but orderly. The dominant speech intention is “report results.” Team members typically use statements like “Blood’s here,” or “Here’s the blood,” to announce the arrival of blood products. The desired outcome includes informing the team lead about the availability of blood products.

The non-routine model for this activity represents scenarios where the arrival of blood products is delayed. It differs from the routine model in four elements: no tools are used, the room (community) is controlled and calm as the team waits for blood products to arrive, unable to proceed with their tasks, speech intention shifts to information requests, and the division of labor shifts to the team lead requesting information about the blood status and nurses providing updates. For example, “Do we have our blood yet?” or “Do we have blood to hang?” The nurse, in response, provides information about delays due to the ongoing process of accessing and transporting blood from the blood bank.

For this activity, speech was key in distinguishing between the structured and more reactive environments in the routine and non-routine models. In the routine model, communication is direct and functional, with team members primarily using declarative statements. In the non-routine model, where delays in obtaining blood products are common, the nature of speech transitions from reporting results to actively seeking information. This change in speech intention signals that normal procedures are disrupted and there is a need for more dynamic decision making. Although tools in this context also indicate delays or complications, speech specifically highlights how the team adapts to and communicates during these disruptions.

#### 4.2.4 Setup of the Rapid Infuser:

**Figure 4:**
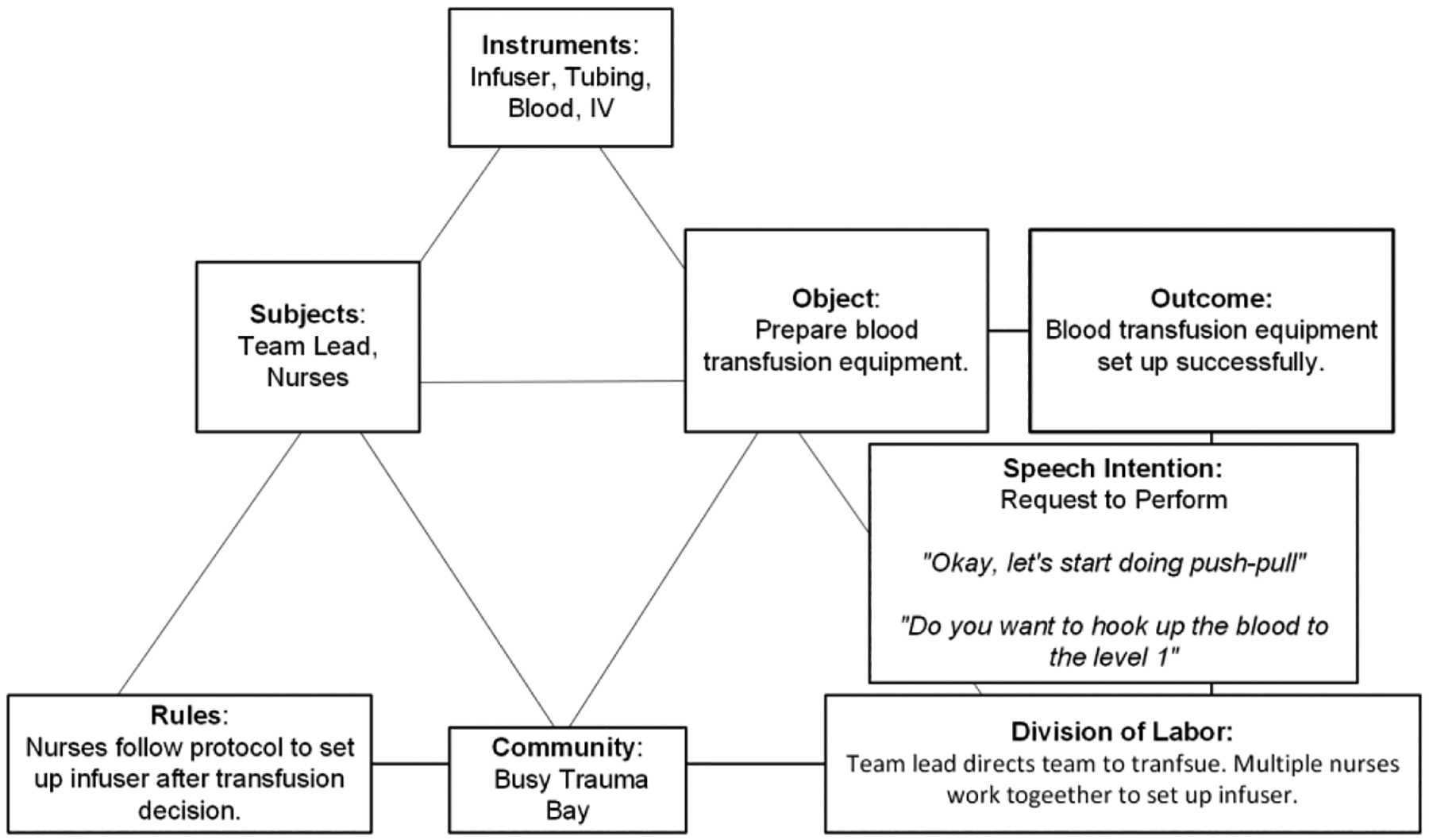
A graphical representation of the activity model for the “setup of the rapid infuser” activity.

We identified only one model for this activity (Figure 4). Our results showed that all 13 activity instances were delayed (Table 4). These delays were attributed to difficulties in setting up the rapid infuser. The subjects are the team lead and nurses. The object(ive) is to set up the infuser to prepare for blood transfusion. To achieve this goal, the team uses the infuser device, tubing, blood products, and the IV catheter. The room (community) is consistently busy, showing increased activity around the patient bed due to the complexity of the setup. The team lead directs the team to transfuse blood and several nurses work together to set up the rapid infuser. The nurses follow the infuser setup protocol after being directed by the team lead. Because the activity requires multiple steps and more than one nurse, it often takes longer to complete. In one delayed case, for example, a nurse attempted to set up the push-pull infuser twice before recruiting help from another nurse. They then attempted to set up the same infuser twice before switching to a level-one infuser and eventually completing the setup. These multiple attempts caused an eight-minute delay in the time to blood transfusion. The dominant speech intention for this activity is “request to perform,” as the team lead instructs the team to perform the setup actions. For example, “Let’s start doing push-pull,” or “Do you want to hook up the blood to level one?” These speech lines showed that the team lead initiated the process, but no speech lines showed a nurse response. The desired outcome is the completed setup of the rapid infuser.

### 4.3 Post-Intervention Analysis of the Acquisition of Blood Products Activity

**Figure 5:**
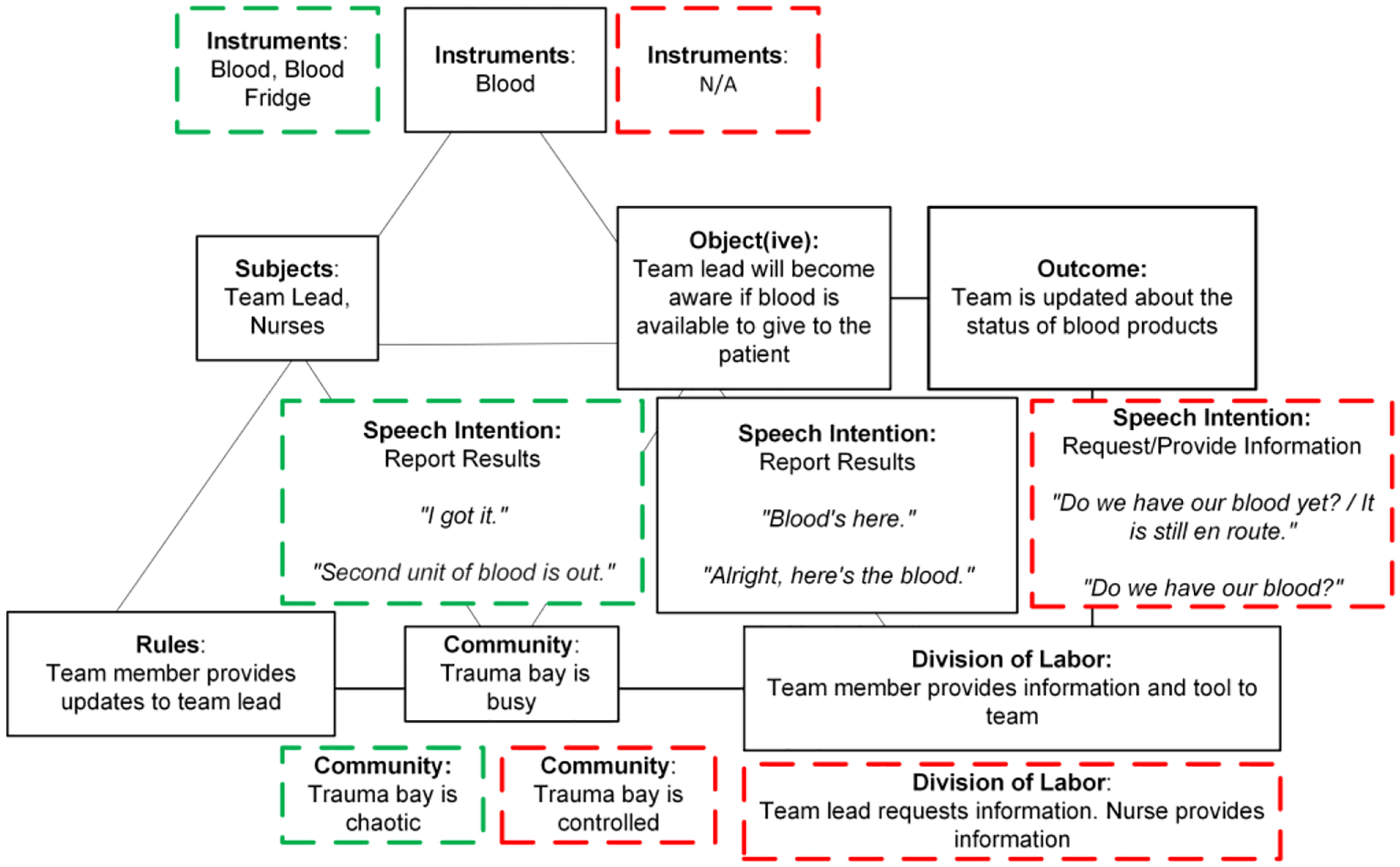
A graphical representation of the post-intervention activity model for the “acquisition of blood products” activity. Deviations from the routine pre-intervention model (shown in the solid black boxes) are depicted in green dashed boxes. Deviations in the non-routine pre-intervention model are shown in the red-dashed lines

Our analysis showed that the introduction of a blood refrigerator had an impact on the “acquisition of blood products” activity model. In all eight post-intervention cases, the activity conformed to the routine pre-intervention model of the activity (Figure 5). The same subjects followed the same rules and division of labor, with team members informing the team lead when blood products arrived. The object(ive) and outcome also remained the same between the pre- and post-intervention models: the acquisition of blood products and notification to the team lead about their availability. The dominant speech intention remained “report results” in the post-intervention model as well. Team members announced the availability of blood by stating “The second unit of blood is out” or “I got it.”

The pre-intervention routine and post-intervention models differ in two elements: a blood refrigerator was added to blood products under instruments and the community (environment) was described as chaotic. This chaotic nature of the room could be explained by the complexity of simultaneously accessing blood products and preparing blood for administration and by the newness of the blood refrigerator, as the team adjusted to directly accessing the instrument rather than waiting for its arrival.

We identified only one delay in obtaining blood products in the eight post-intervention cases, compared to 12 delays (out of 25 activity instances) in the pre-intervention cases (Table 4). This post-intervention delay was due to complicated patient needs that distracted the team from accessing blood products. Our analysis suggests that introducing a blood refrigerator helped reduce delays in obtaining blood products, aligning it closer to a routine process. Although we used a low-tech intervention to evaluate the impact of clinical interventions on the activity, the results highlight the potential of activity theory-based evaluations in identifying and implementing computerized support in complex teamwork.

## 5 DISCUSSION

We next discuss our findings in relation to our three research goals (1) identify activities in the hemorrhage control process that can most benefit from computerized support, (2) determine potential mechanisms for computerized decision support, and (3) examine the effects of an intervention on an activity theory-informed model of a hemorrhage control activity. We also discuss the impact of speech as an additional activity theory prompt on understanding team dynamics during complex activities.

### 5.1 Understanding Complex Teamwork Challenges through Activity Theory

The activity theory framework provided a valuable lens for understanding the challenges in time-critical, medical teamwork by helping us discover a link between variations in activity models. The four delay-causing activities (establishment of vascular access, decision to transfuse blood, acquisition of blood products, and setup of the rapid infuser) had the most variations in activity models, and especially in the division of labor, instruments, community, and speech intentions. We could see how team dynamics and community shifted with delays through changes in individual team member responsibilities and communication. The lack of any instruments suggested an issue or delay was occurring. Shifts in speech intentions—from reports and confirmations to repeated inquiries and need assessments—indicated uncertainty and usually led to changes in the community as well. The rapid infuser setup activity showed a unique pattern. Despite frequent delays, only one activity model emerged, highlighting the challenges in equipment setup rather than team dynamics, decision making, or communication. For the three activities that rarely caused any delays (patient arrival, identification of hemorrhage, and initiation of blood transfusion), the community was usually described as “calm” or “controlled.” The subjects, object(ive)s, rules, and outcomes also changed less frequently than in delay-causing activities. Overall, our findings align with findings from earlier research that identified lapses in communication, decision making, and coordination as the major causes of delays [52][62]. In this paper, however, we went further by identifying the specific kinds of breakdowns in communication (i.e., uncertainty of decision in transfusion decision activity vs. uncertainty of status in the vascular access establishment activity) and how these breakdowns affected other features and elements of the activity.

### 5.2 Identifying CSCW Interventions and Their Mechanisms through Activity Theory

The activity theory approach also allowed us to identify routine and non-routine scenarios within the activities, which in turn helped us identify opportunities for computerized support and appropriate mechanisms for that support. For each delay-causing activity, we identified contradictions between the routine and non-routine models that suggested areas for intervention. We observed that the activities with variations in speech intention and division of labor elements were best suited for computerized support. For example, in the non-routine model for establishing vascular access, the team must first assess the need for IV access and engage in discussion before it can proceed with the activity. Similarly, delays associated with obtaining blood products or proceeding with transfusion after decision manifested through repeated information requests and other communication lapses about blood status and decision to transfuse. Prior studies have also found similar communication breakdowns in complex teamwork. For example, in one case, researchers observed repetitive discussions about the need for blood as different providers arrived before and throughout the procedure, delaying the time to blood transfusion [29]. We identified two major types of CSCW interventions that could resolve the observed contradictions: (1) real-time status displays and (2) computerized and AI-based recommendations.

The uncertainty in the non-routine models highlights a potential need for supporting team situational awareness. A real-time information display indicating the location and status of the patient’s vascular access points or the availability of blood products in real-time could address this uncertainty, limiting confusion about the available access or blood. Clear and real-time communication of the decision to transfuse blood to all subjects involved in the activity could eliminate the issue of not hearing or being unaware of the decision. Introducing real-time status displays to address the challenges of maintaining awareness in complex teamwork has been common in CSCW and HCI (e.g., [4][21][43][53]). For instance, a display showing patient vital signs, the present staff members, a log of orders, administered medication and tests, and a visual timeline of key events was found successful in increasing the team’s situational awareness during emergency department resuscitations [53]. In our case, even a simple display showing the decision and the status of IV access and blood products would likely affect the activity system. This display would affect the activity model by shifting the division of labor back to the routine model where the team lead requests the start of the transfusion protocol and team members follow the instructions.

Computerized and AI-based recommendations in the form of decision-support tools and alerts could also resolve the identified contradictions. A decision support tool that leverages patient data, such as vital signs and reported injury mechanisms, could predict the need for blood transfusion or additional vascular access points. Recommender systems have been found effective in other medical settings by augmenting decision making (e.g., [10][31][65]). Introducing this type of decision support to augment decisions about blood transfusion could reduce the team lead’s cognitive load by providing evidence for and against the intervention based on past cases. However, introducing AI-based tools in time- and safety-critical medical settings requires careful consideration to avoid issues such as over-reliance on the system, the risk of bias in recommendations, and the need for robust validation. Ensuring these tools are thoroughly tested and integrated into existing workflows is essential to maintaining patient safety and effectively supporting team dynamics [66]. Using activity theory to identify specific points in the workflow where a recommendation could be introduced could provide a structured approach to integrating these systems into clinical practice.

Not all activities equally benefit from computerized decision support. As shown in our study, a low-tech intervention like the strategic placement of a blood refrigerator within the trauma bay reduced wait times and minimized communication overhead [61]. Similarly, the absence of contradicting models for the rapid infuser setup suggests that computerized support would not be appropriate for this activity. The activity model did not show significant communication or decision-making issues, but rather highlighted the lack of skills and challenges in the physical aspects of the activity. In this case, an intervention could focus on enhancing training and instructional methods for using the rapid infuser. Our approach here is similar to an activity theory-informed ethnographic study of intensive care units (ICUs) [40]. This prior study identified limited bed availability (tools) and inadequate information transfer to acute care bedside nurses about ICU patients (rules). Based on these limitations, the study recommended improved protocols and education as interventions rather than computerized support.

### 5.3 Introducing Speech to the Activity Theory Model

Speech, a key element in human-human interaction, offers insights into how individuals communicate their intentions within an activity [57][58]. Because speech can influence the dynamics and complexity of trauma resuscitation, it was critical in emphasizing the interdependence of various system elements within the activity theory models [45]. We propose that including speech intention in an activity theory informed analysis can help uncover the nuances in teamwork and how a team achieves the goals of an activity.

In our study, integrating speech as a component within activity theory models provided a better understanding of the dynamics within a team, particularly for the division of labor, object(ive), and outcome prompts. We highlighted this impact of speech intention on the activity model by placing the speech intention box between the division of labor, object(ive), and outcome. The speech intention component is also connected to subjects and community, as speech also tends to affect these elements. By analyzing the utterances and intentions, we could pinpoint team communication breakdowns that otherwise might have remained hidden. Different verbal cues indicated the progress of tasks and inquiries about task status helped identify delays or ongoing work within the activity.

Adding speech intention also helped discern the differences between the routine and non-routine models in the four delay-causing activities. For example, for establishing vascular access activity, the speech intention “report results” is typically aligned with routine, procedural activities where the goal is to convey the results or status of a task. This speech intention showed that specific tasks had been completed and reported to the team lead (division of labor). The speech intention also showed that the goal of the activity had been achieved (outcome). In contrast, the “assess need” speech intention was associated with cases that followed the non-routine model. These cases had a shift in the division of labor, where the team lead and nurses assessed whether an activity was needed. Similarly, in the transfusion decision activity, the speech intention influenced the object(ive), as the team goal shifted from communicating a decision to determining whether a blood transfusion was needed. Speech intentions can signal the start and end of specific actions while also alerting the team to deviations, which is critical for timely interventions in dynamic environments like trauma resuscitation.

Including speech intentions provided additional insights into potential computerized support mechanisms that could help reduce delays in critical activities. By examining disruptions in activities, such as the lack of awareness about the status of IV access or uncertainty about transfusion decisions, it becomes clear that communication breakdowns are a significant issue. Computerized support that offers real-time status updates and display decisions to the team could mitigate these breakdowns. This insight is vital for designing systems for complex teams in high-pressure environments, where excessive alerts or unnecessary information can obstruct teamwork instead of facilitating it [49].

Finally, integrating speech intention enhances activity theory analysis by detailing the impact of speech on activity coordination and completion. Within the medical domain, we use speech intention to understand how the goal of an activity is achieved and how it can affect the system. Domains such as education [12][23][30] could also benefit from including speech intention in their activity theory-informed analysis by adding nuance in the division of labor and outcomes of the activity, further informing the design of support tools. Focusing only on speech intentions may lead to an oversimplification of complex interactions. Future work should include other communication modalities for a more comprehensive insight into the impacts of communication on activity. Even so, analyzing speech intention within a holistic approach such as activity theory can lead to a better understanding and capturing of team interactions and their impact on activity outcomes.

### 5.4 Effects of a Low-Tech Intervention on an Activity Theory-Informed Model

Activity theory suggests that any change in the instruments within an activity model can transform the overall activity [16]. During our study period, a low-tech intervention—a blood refrigerator—was introduced to address delays in obtaining blood products for hemorrhage control. We used this opportunity to study how an activity-theory-informed model of a hemorrhage control activity changes after an intervention. Because we first developed the pre-intervention routine and non-routine models for the “acquisition of blood products” activity, we could study the effects of the intervention by comparing the initial models to the model developed from the post-intervention cases. Our analysis showed that these cases generalized into an activity model with the same prompts as the pre-intervention routine model, but with two key differences. First, a blood refrigerator was added to blood products under instruments in the post-intervention model, reflecting the intervention. Second, the community in the post-intervention was described as more chaotic, likely due to the novelty of the instrument and the team’s adaptation process. Overall, introducing a new instrument shifted the post-intervention activity model towards the pre-intervention routine model, resolving the contradictions between the two models.

With this finding, we contribute to an evaluation framework for comparing pre- and post-intervention activity models informed by activity theory to assess the effects of interventions on teamwork. Prior medical studies have quantitatively evaluated the effects of interventions [44][50][61], while the activity theory was used to analyze activities after introducing a new technology in a classroom setting [23][42]. This framework for qualitatively assessing the impact of interventions through activity theory can show not just the post-intervention improvements in activity performance but also the subtler shifts in team dynamics, communication, and workflow. By analyzing all activity elements, we can identify whether the intervention addressed the identified contradictions within the activity system.

## 6 Conclusion & Future Work

Our study makes several contributions to CSCW and socio-technical systems research. Through an activity-theory informed analysis of a complex medical process, we (1) identified activities that could benefit from computerized support, (2) considered potential mechanisms for computerized support and (3) showed the potential of using activity theory-based evaluations in assessing the impact of clinical interventions on overall activity performance. We also proposed integrating speech intention into the activity theory analysis of complex teamwork after showing how the inclusion of speech led to a better understanding of team dynamics and changes in the activity systems. Unlike prior HCI and CSCW studies that determined the information types for real-time displays based on user-centered research, we analyzed speech intentions, division of labor, instruments, community and other activity elements to pinpoint the information that was needed for effective teamwork. When combined with user research for validation, this targeted and contextually grounded approach to designing computerized interventions could provide a valuable tool for designing computerized support in time-critical settings. Our future work will investigate how implementing the identified computerized interventions affects the activity theory models in hemorrhage control and other complex team processes to further evaluate our approach and its value for CSCW research. Additionally, we will empirically validate these proposed interventions through user studies, ensuring their effectiveness and applicability in real-world settings.

## Figures and Tables

**Table 1:** Classification of speech intentions in trauma resuscitation teamwork [67] applied in this study.

Intention Label	Definition
Assess need for an activity	Team members discuss the results of assessment activities or the outcomes of prior actions to decide whether to perform an intervention.
Assess results of an activity	The team assesses the activity results after completing an activity.
Confirm information receipt or request	A team member indicates that they received the information or heard a request.
Provide information	A team member provides information such as an answer to a request for information, clarification, or feedback to the team.
Report progress of an activity	Team members continuously communicate their activity progress during multi-step activities that take longer to perform.
Report results of an activity	A team member reports the numerical results of an assessment activity or the result of an intervention.
Request action	A team member asks another member or the room to perform or modify an activity. Requests for action also include requests to start or stop an activity, to change volume, or to terminate an activity.
Request information	A team member requests information or clarification about the activity.

**Table 2:** Summary of seven hemorrhage control activities.

Activity	Description
Patient Arrival	Prehospital providers transmit critical information about the patient’s condition and treatments given during transport. The trauma team uses this report to prepare the required resources and equipment.
Identification of Hemorrhage	Trauma teams check for active bleeding during the primary survey and monitor the patient’s blood pressure and pulse throughout the case. Identification of hemorrhage requires teams to use vital sign trends, patient injury characteristics, imaging, and lab results.
Decision to Transfuse Blood	Deciding whether to transfuse blood involves considering the risks and ensuring clear communication among team members to prevent delays. Delays can happen when the decision is not heard, not understood, or not implemented, even if it’s understood.
Establishment of Vascular Access	Securing vascular (IV) access is critical for transfusion, with attempts occurring at different phases of the process. IV methods include peripheral intravenous, central venous, and intraosseous catheters. Delays in establishing IV access can prolong the time to transfusion.
Acquisition of Blood Products	Trauma teams use different strategies to obtain blood products, such as onsite storage or transportation from a blood bank. The chosen method must balance speed with cost considerations. Efficient requests and acquisition of blood are necessary to ensure timely transfusion.
Setup of the Rapid Infuser	Rapid infusers are one type of device used to rapidly infuse blood into patients. Although some manual methods can temporarily increase infusion rates, they are less effective and cannot warm the blood. Proper setup and familiarity with rapid infusers require regular practice to maintain competency.
Initiation of Blood Transfusion	The final step is to initiate blood transfusion using the established IV access and prepared blood products. Additional blood products may be needed to stabilize the patient’s hemodynamic status, requiring efficient repetition of previous activities to avoid further delays.

**Table 3:** Activity theory analysis process for 17 resuscitation cases with hemorrhage control, before a blood refrigerator was introduced.

Hemorrhage Control Activity	Initial AT Models	Standardized AT Models	Generalized AT Model Variations
Patient Arrival	5	3	-
Identification of Hemorrhage	6	4	-
Decision to Transfuse Blood	15	10	2
Establishment of Vascular Access	10	6	2
Acquisition of Blood Products	11	7	2
Setup of the Rapid Infuser	4	2	1
Initiation of Blood Transfusion	6	4	-

**Table 4:** Data summary for 17 resuscitation cases with hemorrhage control.

Activity	Delay Causing Y/N (n)	Activity Occurrences	Avg. Duration of Routine Activities	Avg. Duration of Non-Routine Activities
Patient Arrival	N	17	02:01	02:48
Identification of Hemorrhage	N	15	08:26	14:17
Decision to Transfuse Blood	Y (8)	20	04:54	09:00
Establishment of Vascular Access	Y (9)	24	09:16	11:40
Acquisition of Blood Products	Y (12)	25	10:41	17:18
Setup of the Rapid Infuser	Y (11)	11	13:22	19:36
Initiation of Blood Transfusion	N	17	21:27	33:12

## Box

CCS Concepts: • **Human-centered computing** → Collaborative and social computing theory, concepts, and paradigms → Computer-supported cooperative work
